# Improvement of Lipoplexes With a Sialic Acid Mimetic to Target the C1858T *PTPN22* Variant for Immunotherapy in Endocrine Autoimmunity

**DOI:** 10.3389/fimmu.2022.838331

**Published:** 2022-03-09

**Authors:** Andrea Arena, Eugenia Belcastro, Francesca Ceccacci, Stefania Petrini, Libenzio Adrian Conti, Olivia Pagliarosi, Ezio Giorda, Simona Sennato, Riccardo Schiaffini, Peng Wang, James C. Paulson, Giovanna Mancini, Alessandra Fierabracci

**Affiliations:** ^1^ Infectivology and Clinical Trials Research Department, Bambino Gesù Children’s Hospital, Istituto di Ricovero e Cura a Carattere Scientifico (IRCCS), Rome, Italy; ^2^ Centro Nazionale Ricerche Institute for Biological Systems (CNR -ISB), Secondary Office of Rome-Reaction Mechanisms c/o Department of Chemistry, Sapienza University, Rome, Italy; ^3^ Research Laboratories, Bambino Gesù Children’s Hospital, Istituto di Ricovero e Cura a Carattere Scientifico (IRCCS), Rome, Italy; ^4^ CNR Institute for Complex Systems, Secondary Office of Rome c/o Department of Physics, Sapienza University Rome, Rome, Italy; ^5^ Diabetes and Growth Pathology Unit, Bambino Gesù Children’s Hospital, Istituto di Ricovero e Cura a Carattere Scientifico (IRCCS), Rome, Italy; ^6^ Department of Molecular Medicine, The Scripps Research Institute, La Jolla, CA, United States; ^7^ Centro Nazionale Ricerche Institute for Biological Systems (CNR-ISB), Area della Ricerca di Roma 1, Monterotondo, Italy

**Keywords:** T1D, functionalized lipoplexes, PEGylated lipid F9, immunotherapy, variant PTPN22

## Abstract

The C1858T variant of the protein tyrosine phosphatase N22 (*PTPN22*) gene is associated with pathophysiological phenotypes in several autoimmune conditions, namely, Type 1 diabetes and autoimmune thyroiditis. The R620W variant protein, encoded by C1858T, leads to a gain of function mutation with paradoxical reduced T cell activation. We previously exploited a novel personalized immunotherapeutic approach based on siRNA delivered by liposomes (lipoplexes, LiposiRNA) that selectively inhibit variant allele expression. In this manuscript, we functionalize lipoplexes carrying siRNA for variant C1858T with a high affinity ligand of Siglec-10 (Sig10L) coupled to lipids resulting in lipoplexes (LiposiRNA-Sig10L) that enhance delivery to Siglec-10 expressing immunocytes. LiposiRNA-Sig10L lipoplexes more efficiently downregulated variant C1858T *PTPN22* mRNA in PBMC of heterozygous patients than LiposiRNA without Sig10L. Following TCR engagement, LiposiRNA-Sig10L more significantly restored IL-2 secretion, known to be paradoxically reduced than in wild type patients, than unfunctionalized LiposiRNA in PBMC of heterozygous T1D patients.

## Introduction

The incidence of autoimmune diseases is increasing worldwide (1). With reference to endocrine autoimmunity, insulin-dependent diabetes mellitus (Type 1 diabetes, T1D) and autoimmune thyroid diseases (ATD) are the most common endocrinopathies. The combination is known as polyglandular syndrome Type 3 variant (APS3v) (2). T1D is caused by the targeted destruction of insulin-producing beta cells by autoreactive T lymphocytes (3) and especially occurs in children below 5 years of age (4). This represents the third most common metabolic disorder after obesity and thyroid dysfunction (5, 6). At present, the only available treatment of the endocrine organ-specific autoimmune disorder is the standard substitutive administration of the deficient hormones, *i.e.*, insulin and levo-thyroxine (L-T4). However, in T1D, insulin administration through multiple daily injections does not reproduce the physiological circadian rhythm of natural insulin production. Therefore, it would be desirable to have an immunotherapeutic strategy that preserves the hormonal cells from the autoimmune attack halting the pathogenetic mechanism of disease (7). While the overall goal is clear, T1D is a very heterogeneous disease, due to the contribution of genetic and environmental factors that can impact the efficacy of an immunotherapeutic strategy. Several approaches targeting the immune system have indeed already been experimented but without success since insulin-independence was not achieved in diabetic patients (8, 9).

In light of the foregoing, ‘personalized medicine’, *i.e.*, tailored approaches should be attempted especially based on disease susceptibility under genetic influence. In this regard we have to emphasize the contribution of the major histocompatibility complex (MHC) and of non-MHC genes (10–13). We recently provided evidence, as envisaged by several reports, that C1858T *PTPN22* gene polymorphism which changes amino acid Arg (R) 620 to Trp (W) (R620W) in the encoded lymphoid tyrosine phosphatase protein (Lyp) (7, 13) could be a relevant target for immunomodulation in the treatment of patients affected by an autoimmune disease harboring the variant (7, 14). In this regard, we demonstrated the possibility to achieve target down-modulation of variant *PTPN22* gene by delivering siRNA with liposomes (lipoplexes) to peripheral blood mononuclear cells (PBMC) *in vitro* (7).

Strategies can be improved for targeted delivery of lipoplexes to specific immunotypes playing a major role in the autoimmune disease pathogenesis. On a theoretical basis this can be unraveled by using monoclonal antibodies that drive nanoparticles to T or B lymphocytes through FDA (Food and Drug Administration) approved humanized monoclonal antibodies (MAbs). In recent years the sialic acid-binding immunoglobulin (Ig)-like lectins namely Siglec family of endocytic receptors have been shown to have a restricted expression pattern and function on white cells of the peripheral blood thus capable of modulating innate and adaptive immune responses (15, 16) thus affecting self/non-self discrimination. Thus, they are attractive molecular targets for the treatment of immune-mediated disorders such as autoimmune conditions (17). This led to the development of high avidity glycan mimetic ligands that could selectively target individual Siglecs (17). Of particular interest for this work is that one ligand F9 was specific for Siglec-10 (Sig10L) and when coupled to PEGylated lipid and incorporated into liposomal nanoparticles, upon mixing with PBMC, they bound specifically to a unique monocyte subset having high Siglec-10 expression (17). As regard, of note, loss of function mutations in the human sialic acid acetylesterase (SIAE) gene, responsible for a reduced expression of sialic-acid ligands for Siglec-2 and Siglec-10, were detected in several autoimmune conditions, namely, T1D, polyendocrine syndromes and rheumatoid arthritis (RA) (18–20). Siglec-2 and Siglec-10 also act as checkpoint inhibitors on B cell receptors thus regulating B cell tolerance (21). Their reduced synthesis was hypothesized to lead to uncontrolled B cell activation and systemic autoimmunity (21, 22).

In light of the foregoing we attempted to improve the delivery of lipoplexes targeting C1858T *PTPN22* gene variant (LiposiRNA or L-siRNA (DMPC/**2**/siRNA), *vide infra*) by functionalization with the Sig10L sialic acid mimetic (SAM) to achieve selective immunomodulation in Siglec-10 expressing immunocytes for personalized immunotherapy in patients affected by endocrine autoimmunity with special reference to T1D.

## Materials and Methods

Additional details are provided in the **Supplementary Materials** section (S).

### Study Population

The study population included 13 patients affected by endocrine autoimmunity recruited at the Endocrinology Division, Bambino Gesù Children’s Hospital. All patients were heterozygous (HET) carriers of the C1858T *PTPN22* variant. Presence of the *PTPN22* C1858T variant was evaluated according to previously established protocols (7). Twelve patients were affected by T1D eventually associated with other autoimmune disorders (**Table 1**), *i.e.*, thyroiditis (patient (Pt) 1) and celiac disease (Pt 11, 12). One patient (Pt 13) was affected by Graves’ disease. All patients were recruited during long-term disease. The mean age at referral of patients was 16.84 years (age range 6.35–28.65; 4 women, 9 men) (**Table 1**). The mean age at onset of disease was 8.55 years (age range 4.82–12.86) and the mean disease duration was 8.20 years (age range 0.47–20.4). Demographic and clinical characteristics of patients are shown in **Table 1**.

**Table 1:** Demographic, genetic and clinical characteristics of patients of the present study.

Patient (Pt)	Gender	Age of disease onset (Years)	Age at referral (Years)	Duration of Disease (Years)	Autoimmune disorders	*PTPN22* genotype	HbA1c at referral	Treatment
1	F	8.25	18.84	10.59	T1D, Thyroiditis Hypereosinophilia	1858C/1858T	**79**	MDII
2	M	10.71	13.12	2.41	T1D	1858C/1858T	**67**	MDII
3	M	12.86	13.94	1.09	T1D	1858C/1858T	**60**	MDII
4	M	11.22	14.23	3.01	T1D	1858C/1858T	**54**	MDII
5	F	8.21	28.65	20.44	T1D	1858C/1858T	**66**	CSII
6	F	6.69	10.78	4.09	T1D	1858C/1858T	**58**	CSII
7	M	5.88	6.35	0.47	T1D	1858C/1858T	**68**	MDII
8	M	5.59	9.42	3.83	T1D	1858C/1858T	**74**	MDII
9	M	9.15	20.61	11.46	T1D	1858C/1858T	**61**	CSII
10	M	8.94	23.98	15.04	T1D	1858C/1858T	**73**	CSII
11	F	10.23	23.03	12.81	T1D, Celiac disease	1858C/1858T	**81**	MDII
					Turner syndrome			
12	M	4.82	18.01	13.19	T1D, Celiac disease	1858C/1858T	**65**	MDII
13	M		17.94		Graves’ disease	1858C/1858T		Methimazole

Glycated hemoglobin (HbA1c, reference values 20.0–38.0 mmol/mol). Pathological values are highlighted in bold. MDII, Multiple Daily Insulin Injection; CSII, Continuous Subcutaneous Insulin Infusion.

The sera of patients were assayed for glutamic acid decarboxylase GADA (isoform 65), tyrosine phosphatase-related islet antigen 2 (IA2) antibodies (Abs) and insulin IAA Abs by radioimmunoassay, for Abs to thyroglobulin (Tg), thyroperoxidase (TPO) and transglutaminase (tTGA) by chemiluminescence (ADVIA Centaur analyzer, Siemens Healthcare, Germany) and to parietal cells (PCA) by indirect immunofluorescence (IFL). All recruited patients were unrelated. All subjects entered the study after written informed consent was obtained. The investigation was approved by the local Institutional Review Board (IRB) of Bambino Gesù Children’s Hospital, which regulates human samples usage for experimental studies (Study protocol no. 2135_OPBG_2020); all procedures followed were in accordance with institutional guidelines. The informed consent was obtained from the next of kin in case of children. Consent on behalf of children was written. Participant consent was recorded using a paper-based inventory system. The IRB approved the consent procedure.

### Liposomes Preparation and Characterization

Liposomes composed of 1,2-dimyristoyl-*sn*-glycero-3-phosphocholine (DMPC) and gemini amphiphile *2R,3S*-2,3-dimethoxy-1,4-bis(N-hexadecyl-N,N-dimethylammonium)-butane dibromide, 2 (23), were prepared as previously reported (7, 14).

Moreover liposomes functionalized with the Siglec-10 ligand F9 (Sig10L) were prepared following the same procedure reported in the literature, involving coupling the ligand to PEGylated lipid (F9-PEG-lipid) to facilitate incorporation into liposomes and lipoplexes (17). Liposomes functionalized with F9-PEG-lipid (Lipo-Sig10L) were loaded with lipophilic fluorescent PKH26 dye and 1,2-dimyristoyl-sn-glycero-3-phosphoethanolamine-N-(7-nitro-2-1,3-benzoxadiazol-4-yl) ammonium salt (NBD-PE) for flow cytometry (FACS) and confocal microscopy analysis (*vide infra*). The size distribution and stability over time of liposomes and lipoplexes were investigated by dynamic light scattering (DLS) measurements. Circular dichroism spectroscopy (CD) spectra were also recorded on lipoplexes to further check the stability over time. All the details of preparation and characterization are described in the **Supplementary Materials** (**S1-S3, Supplementary Materials**).

### Circular Dichroism Spectroscopy and Dynamic Light Scattering Analysis

CD spectra and DLS measurements (14) are provided in the **Supplementary Materials** (**S2**, **S3**) (7).

### siRNA Design

Authentic siRNA sequences were designed to specifically target C1858T *PTPN22* gene variant (Rosetta Inpharmatics, Sigma-Aldrich Chemical Co., Saint Louis, MO, US) as previously described (7) (Italian Patent 102018000005182 released on 26.6.2020; Europe, USA and China extended PCT/IT2019/050095 filed on 8.5.2019, Inventor: Dr. Alessandra Fierabracci).

### Custom Liposome Transfection Protocol of PBMC

Liquid-nitrogen frozen PBMC from patients were quickly thawed in pre-warmed RPMI complete medium (Gibco™ RPMI 1640 Medium, ThermoFisher Scientific, Waltham, MA, USA) supplemented with 10% fetal bovine serum (FBS, GE Healthcare, Life Sciences, UT, USA), L-glutamine (2 mM, Euroclone, Milan, Italy) and 1% penicillin/streptomycin (Euroclone) according to established protocols (7, 14, 24). Cells were centrifuged at 1,200 rpm for 5 min at room temperature (RT) and seeded in 48-well plates (flat bottom, Falcon, Corning, NY, USA) at a density of 1.5 × 10^6^ per well in a final volume of 250 μl of FBS-free RPMI 1640 medium containing L-glutamine (2 mM) and treated with different doses of lipoplexes LiposiRNA-Sig10L (L-siRNA-Sig10L) complexes (80 and 100 pmols of siRNA) (7). After approximately 16 hours (h) (overnight, O/N) of transfection, cells were washed by 1,200 rpm centrifugation for 5 min at RT. The cells were subsequently replated in 48-well plates in a final volume of 250 μl of complete RPMI medium. Cells were further incubated for 24 or 48 h at 37°C in a 5% CO_2_ humidified atmosphere.

### RNA Extraction and Quantitative Real Time-PCR Analysis

Total RNA was isolated from samples with TRIzol™ Reagent (Invitrogen, Life Technologies Corporation, Carlsbad, CA, USA) according to the instructions of the manufacturer. After *in vitro* reverse transcription (500 ng) with the High-Capacity cDNA reverse transcription kit (Applied Biosystems, Foster City, CA), quantitative Real-Time PCR (rtq-PCR) was performed using QuantStudio™ 12K Flex Real-Time PCR System (Applied Biosystems) and Power SYBR Green PCR Master Mix (Applied Biosystems) according to established protocols (7) (Italian Patent 102018000005182, PCT/IT2019/050095).

### Confocal Microscopy Analysis and Toxicity Assay

Protocol details are found in the **S6**, **S7** sections of the **Supplementary Materials**.

### Functional Assay Studies Using LiposiRNA-Sig10L Lipoplexes

Evaluation of IL-2 concentration was assayed in supernatants of patients PBMC transfected O/N with L-siRNA and L-siRNA-Sig10L at the dose of 80 pmols of siRNA, then treated with Dynabeads Human T-activator CD3/CD28 beads (Invitrogen). After the O/N transfection, cells were washed by centrifugation, seeded 2.5 × 10^5^ per well in 96-well flat bottom plates in complete RPMI medium, then activated with anti-CD3/CD28 beads at the bead-to-cell ratio of 1:50 and then incubated at 37°C in a humidified atmosphere containing 5% CO_2_ for 5 days (7). At the end of the incubation period, supernatants and cells were collected and separated by centrifugation at 1,200 rpm for 5 min. Supernatants IL-2 concentration was estimated by means of the human IL-2 ELISA development kit (Elisa^Pro^ Kit, Mabtech, Nacka Strand, Sweden) according to the instructions of the manufacturer. Plates were read at 405 nm by Bench-mark Plus microplate spectrophotometer (Bio-Rad, CA). The evaluation was performed on at least triplicate biological determinations. Demographic and clinical characteristics of the patients are shown in **Table S2**.

### Statistical Analysis

Values are expressed as means ± SEM. Differences between each test condition and the control condition were assessed by one-way ANOVA analysis of variance followed by Bonferroni multiple comparison test, for the L-siRNA internalization and toxicity experiments. Differences between each test condition and the control condition were assessed by Kruskal–Wallis analysis and Mann–Whitney t-test, for the L-siRNA efficacy experiments. Statistical study was performed with GraphPad Prism version 7 (GraphPad software, San Diego, CA, USA). Difference was considered significant when the P-value was less than 0.05.

## Results

### Assessment of Size and Polydispersity Index of Liposome and Lipoplexes by DLS Analysis

DLS analysis showed that both liposomes and lipoplexes, in the presence or in the absence of F9-PEG-Lipid, have a small hydrodynamic diameter (∼ 60 nm, details are reported in **Supplementary Materials**, **Table S1**) and are stable up to 48 h. The presence of siRNA at vesicle surface causes a slight decreasing of the overall size of the DMPC/**2**/F9-PEG-Lipid/siRNA lipoplexes, which can be attributed to the presence of PEG chain and to a reduction of the hydration shell thickness.

### CD Evaluation of siRNA Stability in Lipoplexes

CD measurements were recorded for both lipoplexes (DMPC/**2**/siRNA and DMPC/**2**/F9-PEG-Lipid/siRNA) and compared to that of siRNA that remains free in HEPES/EDTA solution. CD bands of siRNA in both lipoplexes feature the same shape of free siRNA. However, a reduction of bands intensity is observed together with a bathochromic shift, these features being more evident in the presence of F9-PEG-Lipid (**Figure S1**). All details and CD spectra are reported in the **Supplementary Materials**.

### LiposiRNA-Sig10L Lipoplexes Are Effectively Internalized in PBMC

**Figure 1:**
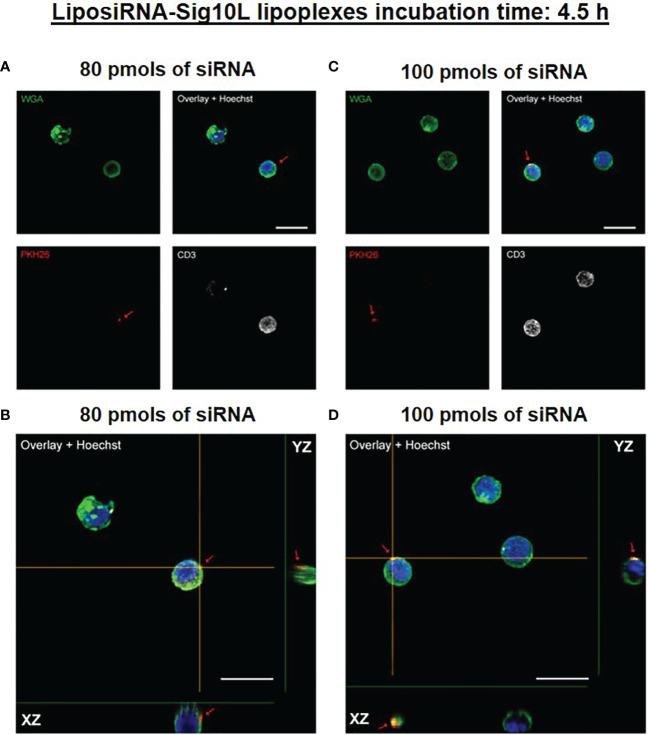
PKH26-labeled LiposiRNA-Sig10L (L-siRNA-Sig10L) internalization in HD PBMC after 4.5 h of incubation. Confocal microscopy images show the internalization of LiposiRNA-Sig10L particles (PKH26 positive dots, arrows) in CD3^+^ (white) cells among HD PBMC after 4.5 h of treatment with lipoplexes at 80 **(A)** and 100 **(C)** pmols of siRNA. Confocal Z reconstructions show the distribution of PKH26-labeled LiposiRNA-Sig10L particles beneath the cell membrane in CD3^+^ lymphocytes (arrows) upon treatment with different doses of lipoplexes 80 **(B)** and 100 **(D)** pmols of siRNA. Cell membrane and nuclei are stained with WGA (green) and Hoechst dye (blue) respectively. Bar: 10 μm.

**Figure 2:**
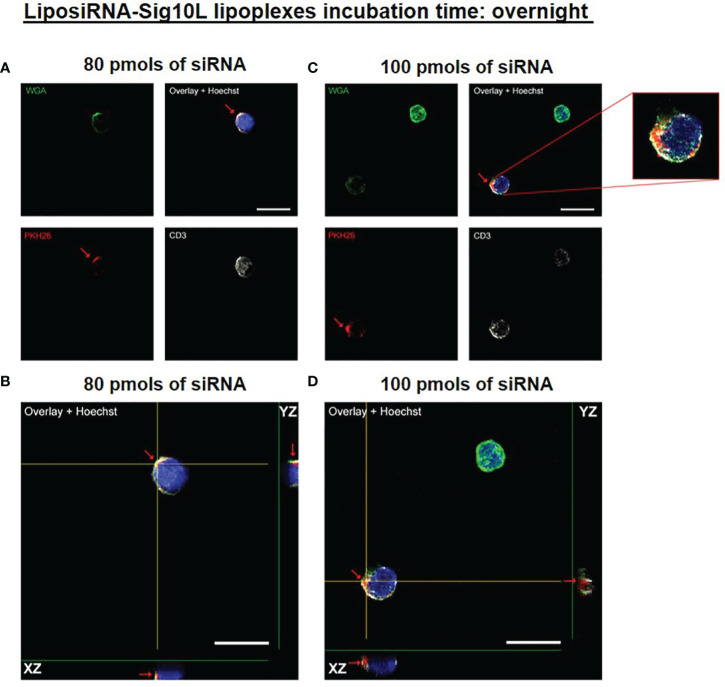
PKH26-labeled LiposiRNA-Sig10L internalization in HD PBMC after O/N of incubation. Confocal microscopy images show the internalization of LiposiRNA-Sig10L particles (PKH26 positive dots, arrows) in CD3^+^ (white) cells among HD PBMC after O/N treatment with lipoplexes at 80 **(A)** and 100 **(C)** pmols of siRNA. Confocal Z reconstructions show the internalization of PKH26-labeled LiposiRNA-Sig10L particles in CD3^+^ lymphocytes (arrows) upon treatment with different doses of lipoplexes 80 **(B)** and 100 **(D)** pmols of siRNA. Cell membrane and nuclei are stained with WGA (green) and Hoechst dye (blue) respectively. Bar: 10 μm.

**Figure 3:**
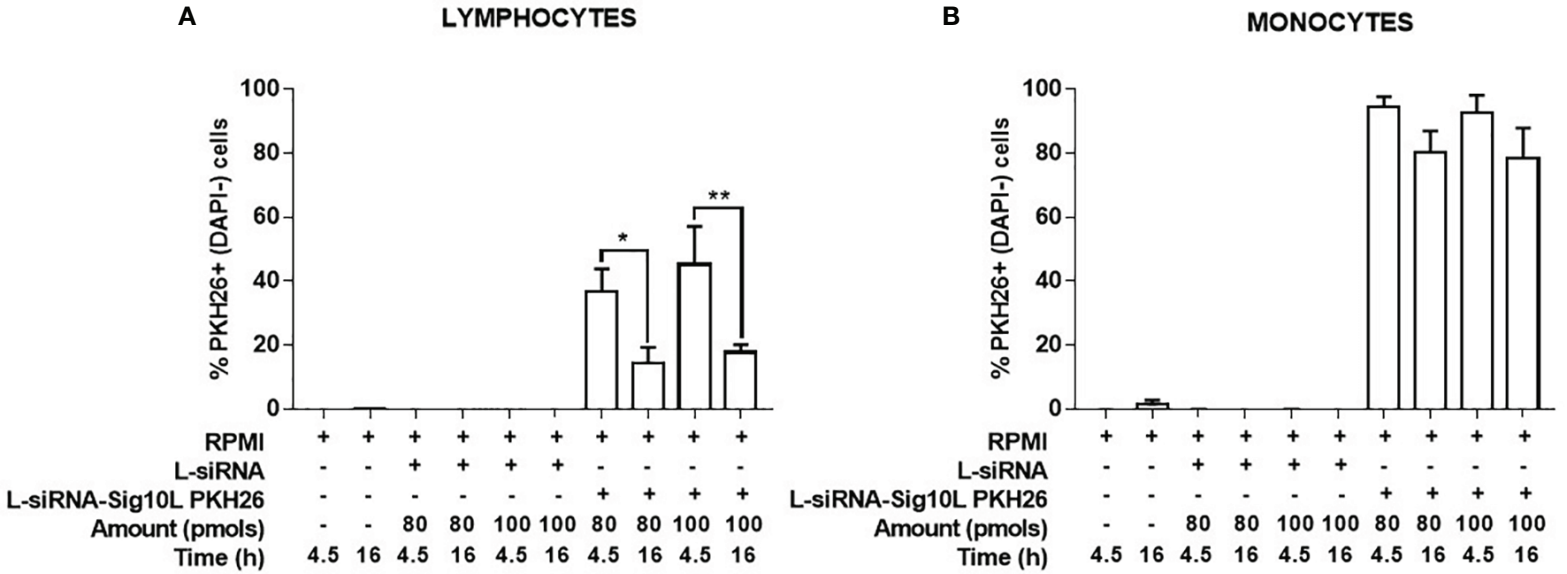
Evaluation of PKH26-labeled LiposiRNA-Sig10L internalization in HD PBMC. Flow cytometry analysis of HD PBMC treated for 4.5 h or O/N with indicated doses of unlabeled LiposiRNA (L-siRNA) or PKH26-labeled LiposiRNA-Sig10L. Histograms show the total percentages PKH26^+^ (DAPI−) lymphocytes **(A)** and monocytes **(B)** upon the indicated treatments performed for 4.5 h and O/N. Gating strategy is illustrated in **Figure S2**. Data are expressed as mean ± SEM of n = 3 HD samples. *p < 0.05, **p < 0.01.

Internalization of PKH26-conjugated LiposiRNA-Sig10L complexes at the doses of 80 and 100 pmols of siRNA in HD PBMC following 4.5 h (**Figure 1**) or O/N (**Figure 2**) incubation was observed. The projections on the X- and Y-axes of the Z-reconstructions of confocal single optical sections showed the distribution of PKH26-labeled LiposiRNA-Sig10L particles beneath the cell membrane in CD3^+^ cells (red arrows) upon treatments at 80 (**Figures 1A, B**) and 100 pmols (**Figures 1C, D**) of siRNA after 4.5 h of incubation. After O/N incubation, the study of the XY-Z orthogonal projections of confocal images allowed clear detection of lipoplexes internalization of PKH26-labeled LiposiRNA particles in the CD3^+^ cells (red arrows, **Figure 2**). Internalization of PKH26-labeled LiposiRNA-Sig10L in HD PBMC was further confirmed by FACS analysis, revealing high percentage of PKH26 positivity into lymphocytes (**Figure 3A**) and monocytes (**Figure 3B**).

### LiposiRNA-Sig10L Lipoplexes Are Not Toxic to PBMC

**Figure 4:**
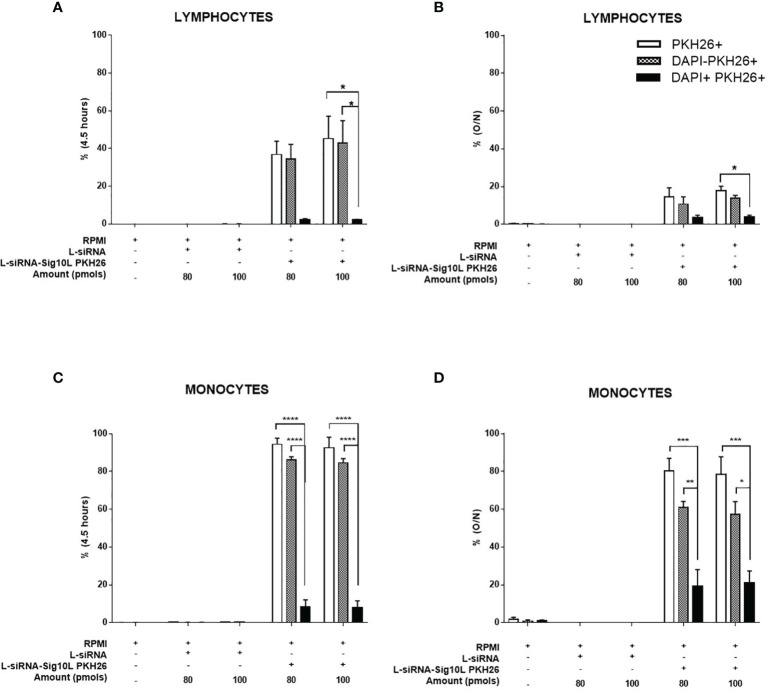
Evaluation of PKH26-labeled LiposiRNA-Sig10L toxicity on HD PBMC. Flow cytometry analysis of HD PBMC treated for 4.5 h or O/N with the indicated doses of unlabeled LiposiRNA or PKH26-labeled LiposiRNA-PEGF9. Histogram shows the percentages of total PKH26+, live PKH26^+^ (DAPI−) and dead PKH26^+^ (DAPI+) among lymphocytes **(A, B)** and monocytes **(C, D)** upon the indicated treatments performed for 4.5 h **(A–C)** and O/N **(B–D)**. Gating strategy is illustrated in **Figure S2**. Data are expressed as mean ± SEM of n = 3 HD samples. *p < 0.05, **p < 0.01, ***p < 0.001, ****p < 0.001.

FACS analysis of HD PBMC treated with different doses of PKH26-conjugated LiposiRNA-Sig10L lipoplexes for 4.5 h (**Figures 4A, C**) or O/N (**Figures 4B, D**) significantly revealed high percentage of PKH26^+^ cells implying relevant transfection efficacy and internalization for both lymphocytes and monocytes. Results showed low percentage of dead cells (PKH26^+^DAPI^+^ cells) (**Figure 4**) indicative of low lipoplexes toxicity (gating strategy is illustrated in **Figure S2**)

### LiposiRNA-Sig10L Lipoplexes Treatment Downregulates *PTPN22* mRNA More Efficiently Than LiposiRNA Lipoplexes

**Figure 5:**
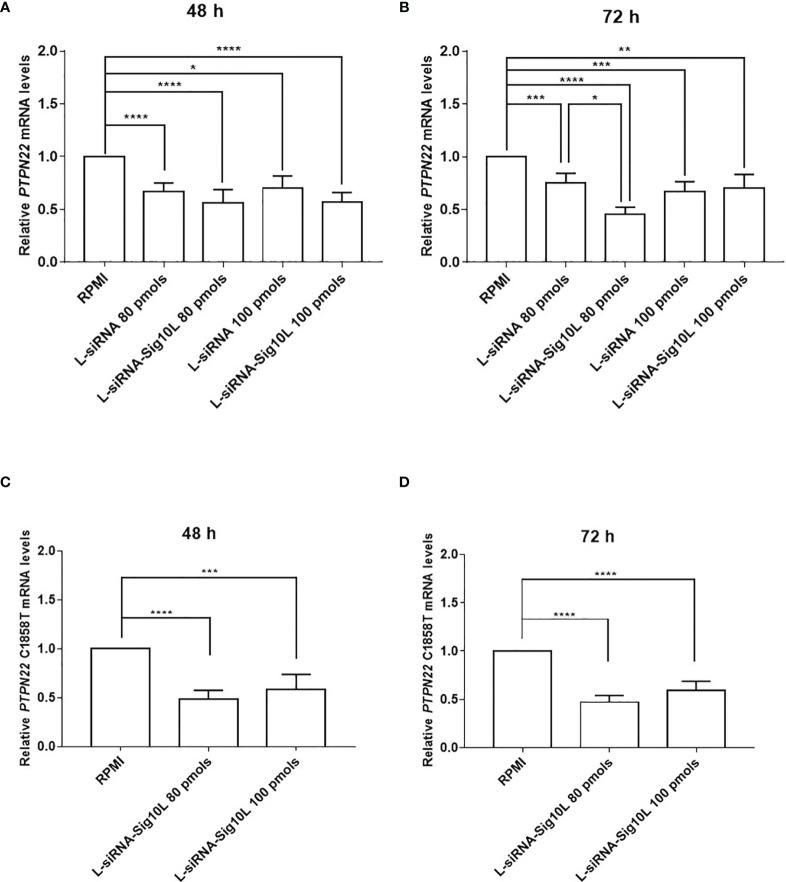
Assessment of LiposiRNA and LiposiRNA-Sig10L efficacy on target *PTPN22* and C1858T *PTPN22* mRNA. Decrease of target *PTPN22* mRNA in HET T1D PBMC analyzed by rtq-PCR upon 48 h **(A)** and 72 h **(B)** of lipoplexes treatment. Rtq-PCR analysis of mRNA from HET T1D PBMC treated with lipoplexes at the indicated doses for 48 h **(C)** and 72 h **(D)** using a set of specific primers to recognize C1858T *PTPN22* mRNA. Values correspond to mean frequency ± SEM of n = 13 **(A, B)** and n = 12 **(C, D)**. *p < 0.05, **p < 0.01, ***p < 0.001, ****p < 0.0001.

PBMC derived from heterozygous C1858T *PTPN22* T1D patients were treated with different doses of lipoplexes (80 and 100 pmols of siRNA) for 48 and 72 h of incubation. mRNA isolated from treated cells was analyzed by rtq-PCR. Results led to a significant decrease in the target *PTPN22* mRNA levels, clearly suggesting the efficacy of the functionalized lipoplexes to downregulate total *PTPN22* mRNA more than LiposiRNA not functionalized respect to RPMI alone (**Figure 5**), while it did not affect the mRNA levels in the wild-type patients (**Figure S3**).

To corroborate these data, after 72 h of incubation at 80 pmols of siRNA a significant reduction of *PTPN22* mRNA levels by LiposiRNA-Sig10L over LiposiRNA was observed indicating a higher efficacy of delivery of the Siglec-10 targeted lipoplexes (**Figure 5B**) (7). No such effects were observed following the treatment of the cells with higher doses of functionalized LiposiRNA-Sig10L (7). To ascertain lipoplexes variant specificity, a second set of primers aimed to solely detect C1858T variant mRNA was used. The rtq-PCR results confirmed the functionalized lipoplexes efficacy to specifically target and downregulate variant C1858T *PTPN22* mRNA respect to RPMI alone at both doses (**Figures 5C, D**).

### LiposiRNA-Sig10L Lipoplexes Restore Lyp Biological Activity at Higher Efficiency Than LiposiRNA

Autoimmune disease associated R620W Lyp is “a gain of function” enzyme variant (25) considering the variant phosphatase as a more potent regulator of T cell signaling paradoxically leading to diminished lymphocyte activation. As previously reported in the literature, a decrease of IL-2 secretion by HET C1858T *PTPN22* PBMC compared to wild type was shown (7). After T cell receptor (TCR) engagement, an increased IL-2 secretion upon lipoplexes treatment respect to untreated cells (RPMI) was observed in HET C1858T *PTPN22* T1D PBMC in comparison to wild type *PTPN22* T1D PBMC (7).

**Figure 6:**
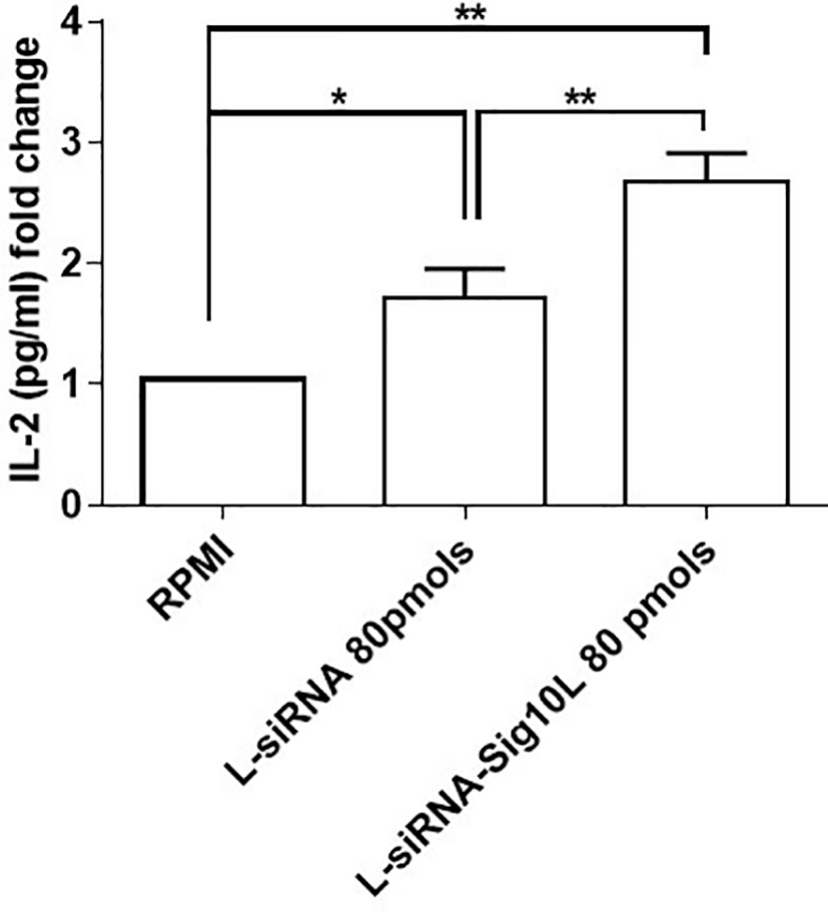
IL-2 detection in supernatants of transfected then anti-CD3/CD28 stimulated PBMC. IL-2 secretion upon 5 days of anti-CD3/CD28 activation (bead to cell ratio 1:50) in culture supernatants of T1D C1858T *PTPN22* HET PBMC transfected O/N with LiposiRNA-Sig10L at the dose of 80 pmols. Data are expressed as mean ± SEM of n = 5 *p < 0.05, **p < 0.0025.

Similarly, current data demonstrated a more significant increase of IL-2 secretion upon LiposiRNA-Sig10L treatment at the dose of 80 pmols of siRNA *versus* untreated cells and not functionalized lipoplexes in HET C1858T *PTPN22* T1D PBMC, using a suboptimal condition for stimulation with anti-CD3/CD28 beads (**Figure 6** and **Table S2**). These data confirmed that the “gain of function” effect of Lyp R620W on TCR signaling^25^ could be restored following treatment with functionalized lipoplexes, by suggesting a possible recovery of normal Lyp regulatory performance.

## Discussion

Experimental evidence suggests that the C1858T single nucleotide polymorphism (SNP) of *PTPN22* (rs2476601) by disrupting binding between the variant Lyp and C terminal Src Kinase (CSK) (26) compromises suppression of T cell activation. Since the T allele binds less efficiently to CSK than the C allele, C1858T T lymphocytes are indeed hyperresponsive and predispose harboring individuals to autoimmunity (26). The variant has also effect on other immunotypes that play a prominent role in different autoimmune conditions as reported for T regulatory cells (Tregs) (27, 28), macrophages (29) and B lymphocytes (24). Further, epidemiological studies confirm that the functional C1858T *PTPN22* SNP is associated with susceptibility to several autoimmune diseases both organ-specific (T1D) and systemic [RA and systemic lupus erythematosus (SLE)] in different populations and that its prevalence is ethnicity-dependent (13, 26, 30). As regard endocrine autoimmunity, in particular the T cell mediated disease T1D, C1858T *PTPN22* polymorphism is responsible for a more severe phenotype in humans with more precocious onset and more rapid decline of beta cell function (7, 31). Consistently, in a model of transgenic R619 non-obese diabetic (NOD) mice generated with the Clustered Regularly Interspaced Short Palindromic Repeats-Caspase 9 (CRISPR/Cas9) technology, T1D onset occurs earlier with higher penetrance and higher autoantibodies titers (32). As regard SLE, B lymphocytes exert a dominant etiopathogenetic role; nevertheless perturbed immunoregulations also underpin an imbalance between CD4^+^ Tregs and T effector (Teff) cells. Indeed, in SLE patients the presence of the risk allele C1858T reproducibly correlated with increased T cell hyperactivity leading to compensatory increased frequencies of thymically-derived FOXP3^+^Tregs. Further in C1858T *PTPN22* SLE carriers enhanced expression of PD-1 was associated with reduced Tregs suppressive capacity and increased autoreactive Teff proliferation contributing to disease flares of clinically active disease (33).

Strategies to improve specific cell delivery of lipoplexes generated against the variant C1858T can employ targeting agents such as FDA approved MAbs. In this regard, anti-CD3 (otelixizumab) (34), anti-CD3 teplizumab (35) or anti-CD20 (rituximab) (36, 37), can be used to functionalize lipoplexes (LiposiRNA-antiCD3, LiposiRNA-antiCD20) and target T and B lymphocytes (37) respectively and also anti-lymphocyte function-associated antigen 1 (LFA-1, CD11a) (38) or anti LFA-3 MAbs (39).

In this manuscript, we explore the possibility to functionalize lipoplexes with SAM PEGF9 (17) that specifically target human immunocytes expressing this Siglec (16). As reported in the literature, Siglec molecules can regulate the immune balance in autoimmunity and cancer conditions (40). The molecule Siglec-10 is an inhibitory receptor broadly expressed on B lymphocytes, monocyte-derived dendritic cells, macrophages (16) and predominantly on NK cells (41). Siglec-10 was also found upregulated on some human activated CD4^+^ T cells, consistent with its homeostatic role of inhibition on T cell activation by affecting the phosphorylation of the TCR-associated kinase ZAP-70 (42, 43). The major findings of the present study indicate the ability of LiposiRNA-Sig10L lipoplexes (at 80 pmols of siRNA) to specifically target and significantly decrease C1858T *PTPN22* mRNA levels in heterozygous T1D PBMC. These data suggest that functionalized lipoplexes are more efficient to downregulate the variant than non-targeted LiposiRNA. It is known from literature that the variant Lyp affects T lymphocytes activation more vigorously than wild type Lyp (25). In a previous work (7), we demonstrated that, following TCR engagement, LiposiRNA treatment restored in PBMC of heterozygous T1D patients IL-2 production which is known to be paradoxically reduced than in wild type T1D patients. Indeed, in the present investigation we verified that treatment of PBMC of heterozygous individuals with LiposiRNA-Sig10L at the indicated dose, following TCR engagement, more significantly restored IL-2 secretion than LiposiRNA. Further insights can be highlighted on the possible effects that functionalized LiposiRNA lipoplexes may cause in Siglec-10 positive activated T cells of T1D C1858T heterozygous patients that are specifically targeted in our system. Indeed in addition to act as a vehicle of siRNA to Siglec-10 positive immunocytes, we can speculate on an agonist effect through F9-PEG-lipid *per se* with the soluble CD52 glycan in suppressing T cell activation (43). As regard high CD52 CD4^+^ T cells represent a subset of Tregs distinct from conventional CD4^+^CD25^+^ Tregs (44, 45). CD52 sequesters the proinflammatory damage associated molecular pattern (DAMP) protein high mobility group box 1 (HGMB1) that in turn engages Siglec-10 (46). Interestingly in response to the autoantigen GAD65, but not tetanus toxoid, percentages of CD52highCD4^+^ T cells are significantly lower in individuals than in patients with overt T1D (43). Nevertheless, the roles of Siglecs and in particular of Siglec-10 remains to be fully elucidated. Siglec-2 and Siglec-10 appear to be involved in B cell tolerance acting as immunosuppressive receptors that specifically counteract B cell receptor (BCR) signaling (47) and B cell proliferation in lymphoma (48). This suggests that in our system, LiposiRNA lipoplexes could also be directed to B lymphocytes. In our laboratory we previously reported altered B cell homeostasis and toll-like receptor 9-driven response in T1D patient carriers of the C1858T *PTPN22* allelic variant (24).

This led to hypothesize a putative effect on B lymphocytes and, on a speculative basis, their applicability to the treatment of B cell-mediated autoimmune conditions opening perspectives in the field of personalized immunotherapies.

On a general ground the approach based on the use of functionalized lipoplexes we need to remark that this is a personalized gene therapy approach of specific immunocytes. This should avoid side effects of general immunosuppression encountered in trials with MAbs immunotherapeutic approaches such as anti-CD3 (49, 50).

In summary the results presented in this investigation highlight the possibility to improve lipoplexes delivery and efficacy in downregulating C1858T *PTPN22* variant mRNA by functionalization with the sialic-acid mimetic PEGF9. We also demonstrated safety *in vitro* of the functionalized preparation and its functional activity, following TCR engagement, being able to significantly restore IL-2 secretion more than unfunctionalized LiposiRNA in PBMC of heterozygous individuals where is known to be paradoxically reduced that in wild type patients. These results open future pathways of preclinical investigations with the aim of estimating the biodistribution of LiposiRNA-Sig10L lipoplexes in C57BL/6 mice and their safety and efficacy in an *in vivo* animal model of disease. As regards, we are generating a transgenic NOD mice harbouring the variant C1858T *PTPN22* (51) in order to achieve this final goal. By LiposiRNA-Sig10L lipoplexes administration in this transgenic mice we will assess the efficacy of the novel drug in delaying the disease onset or halting the disease progression. In case positive safety and efficacy results will be obtained, clinical grade formulations of the new drug will be potentially evaluated in a phase I/II clinical trial in adults patients. The successful results of the trial in adults will be open new pathways to trials in pediatric patients with T1D and APS3v aiming to ascertained the applicability of the novel personalized immunotherapy. This will represent a significant advance beyond conventional substitutive insulin treatment since the immunotherapeutic intervention will halt the pathogenic mechanism thus preserving beta cells from autoimmune destruction in the preclinical period or avoiding the progressive destruction of their reservoir after the disease onset.

## Data Availability Statement

The original contributions presented in the study are included in the article/**Supplementary Material**. Further inquiries can be directed to the corresponding author.

## Ethics Statement

The studies involving human participants were reviewed and approved by the Institutional Review Board of Bambino Gesù Children’s Hospital. Written informed consent to participate in this study was provided by the participants’ legal guardian/next of kin.

## Author Contributions

AF, GM: Conceptualization. AA, EB: Data curation. EB, AA, SP, LAC, EG, SS, and FC: Formal analysis. AF: Funding acquisition. AF, EB, AA, OP, and FC: Investigation. AF, EB, AA, FC, and PW: Methodology. AF: Project administration. AF, RS, EG, SP, PW, and JPC: Resources. EB, AA, SP, LAC, and EG Software. AF, and GM: Supervision. AF, JCP, GM, and FC: Validation. EB, AA, AF, GM, FC, and EG: Visualization. AF, AA, GM, and FC: Writing—original draft. AF, AA, EB, GM, and FC: Writing—review & editing. All authors listed have made a substantial, direct, and intellectual contribution to the work and approved it for publication.

## Funding

This work was supported by the Italian Ministry of Health Ricerca Corrente 2020–2021 and Ricerca Finalizzata RF-2019-12369889 (AF) and the National Institutes of Health grant AI132790 (JP).

## Conflict of Interest

The authors declare that the research was conducted in the absence of any commercial or financial relationships that could be construed as a potential conflict of interest.

## Publisher’s Note

All claims expressed in this article are solely those of the authors and do not necessarily represent those of their affiliated organizations, or those of the publisher, the editors and the reviewers. Any product that may be evaluated in this article, or claim that may be made by its manufacturer, is not guaranteed or endorsed by the publisher.
