# Supplementary material for: Improvement of Lipoplexes With a Sialic Acid Mimetic to Target the C1858T PTPN22 Variant for Immunotherapy in Endocrine Autoimmunity
Source: Front Immunol. 2022 Mar 9;13:838331. doi: 10.3389/fimmu.2022.838331 (PMC8959661; doi:10.3389/fimmu.2022.838331)
Supplement: Supplementary file 1 [file DataSheet_1.docx]

Supplementary Material

**Methods**

*S1, Preparation and characterization of liposome formulations*

Gemini amphiphile 2R,3S-2,3-dimethoxy-1,4-bis(N-hexadecyl-N,N-dimethylammonium)- butane dibromide, **2**, was prepared as previously described^23^ .

Liposomes composed of DMPC (purity*>*99 %, Avanti Polar Lipids Inc. (Alabaster, AL) and **2** in a 1/1 molar ratio were prepared following a previously described procedure^14^. In a round bottom flask proper volumes of a solution of DMPC (in CHCl_3_) and a solution of **2** (in CHCl_3_) were added and the solvent slowly evaporated to obtain a thin film. After 5 hours under high vacuum, the film was hydrated with a buffer solution (5 mM HEPES and 0.1 mM EDTA, at pH 7.4) to have a final dispersion 0.5 mM in DMPC and 0.5 mM in **2**. The dispersion was vortex-mixed, freezed-thawed six times from liquid nitrogen to 40 °C, and finally extruded (10 times) through a 100 nm polycarbonate membrane (Whatman Nuclepore, Toronto, ON, Canada) in a 10 ml extruder (Lipex Biomembranes, Vancouver, Canada), at 40 °C (well above the DMPC transition temperature, 24.2 °C).

In the case of liposomes functionalized with F9-PEG-lipid^17^, this component was added in the film preparation step in a 2.8 % molar percentage with respect to the overall composition.

For fluorescently labeled liposomes, 1,2-dimyristoyl-sn-glycero-3-phosphoethanolamine-N-(7-nitro-2-1,3-benzoxadiazol-4-yl) ammonium salt (NBD-PE-14:0, purity*>*99 %, Avanti Polar Lipids Inc.) was added in the film preparation step at a 0.5 % molar percentage whereas PKH26 dye (ethanolic solution, Sigma-Aldrich Company Co., St Louis, MO, USA) at 0.1 %.

For the preparation of lipoplexes L-siRNA (DMPC/**2**/siRNA) or L-siRNASig10L (DMPC/**2**/ F9-PEG-Lipid /siRNA), a proper volume of a 2.6 μM siRNA solution (diluted from 0.1 mM stock solution in HEPES/EDTA buffer) was added to an equal volume of a liposome solution (at 0.2 mM total lipid concentration) to obtain the following final concentrations: [siRNA] = 1.3 μM, [DMPC] = 50 μM, [**2**] = 50 μM, corresponding to a charge ratio +/- = 2.

Samples were analyzed by circular dichroism spectroscopy (CD) and dynamic light scattering (DLS) after 2 and 48 hours from preparation.

All liposomes and lipoplexes were used for biological experiments at 2 hours from preparations.

*S2, Circular dichroism spectroscopy*

CD spectra were recorded on a Jasco spectropolarimeter J-715 equipped with a Peltier device for the temperature control, using 0.5 cm path length quartz cuvettes. CD spectra are the results of 16 scans in the 330-220 nm spectral range at 20 °C (scanning speed of 100 nm/min, response time of 1 second, resolution of 1 nm). CD spectra were recorded for [siRNA] = 1.3 µM in HEPES/EDTA buffer and for siRNA in lipoplex (DMPC/**2**/siRNA and DMPC/**2**/F9-PEG-Lipid/siRNA) after 3 h and 48 hours from preparations. Results (**Figure S1**) show that the association of siRNA to liposomes causes a reduction of the intensity of CD bands as well as bathochromic shift, more significant in the case of DMPC/**2**/F9-PEG-Lipid/siRNA lipoplexes. After 48 hours a modest further change is observed for DMPC/**2**/siRNA whereas for DMPC/**2**/F9-PEG-Lipid/siRNA lipoplexes CD bands present a more consistent intensity decrease together with the appearance of a shoulder around 282 nm.

*S3, Dynamic light scattering*

DLS was used to determine the hydrodynamic size of liposomal samples at 25 °C. A Malvern NanoZetaSizer apparatus equipped with a 5 mW HeNe laser (Malvern Instruments LTD, UK) and a Peltier system for temperature control were employed.

The intensity scattered by the sample is collected in a backscattering configuration (173 ° angle). The autocorrelation function of scattered intensity as a function of the decay times is analyzed to obtain the distribution of the diffusion coefficients D of the particles, which in turn are converted in a distribution of hydrodynamic radii R_H_ using the Stokes Einstein relationship R_H_ = k_B_T/6пηD, where k_B_T is the thermal energy and η the solvent viscosity.

As preliminary analysis, the cumulant method has been considered to get the average hydrodynamic size RH and the polidispersity index (PDI)^1^. To understand the effect of inclusion of F9-PEG-Lipid and of the interaction with siRNA on liposome size and size distribution, a fine analysis of the size distribution has been performed by NNLS algorithm^2^. By this analysis it is possible to ascertain the possible presence of two or more populations with distinct size. Note that in the intensity-weighted analysis that is commonly employed in DLS data analysis, the obtained RH is biased on larger size, being the scattered intensity proportional to the sixth power of the particle size. With the aim of evaluating the relative abundance of two populations, we have thus performed a number-based analysis. Results are expressed as the average of three different measurements, each measurement was averaged over at least 20 runs (**Table S1**).

DLS measurements on the liposome formulations of DMPC/**2** and DMPC/**2**/F9-PEG- Lipid and on lipoplexes (DMPC/**2**/siRNA or DMPC/**2**/F9-PEG-Lipid/siRNA) have been performed as described in a previous work^3^.

Results of the cumulant analysis indicate that DMPC/**2** liposomes have a hydrodynamic diameter smaller than 100 nm and a low PDI index, while the presence of F9-PEG-Lipid causes a slight increase of both size and PDI. The analysis of the number-weighted size distribution reveals that both formulations have a monomodal size distribution and the average diameter is approximately the same.

Complexation of liposomes with siRNA does not promote any variation in the size of DMCP/**2** liposomes and both average diameter and PDI remains almost identical, as well as their size distribution determined by NNLS analysis (**Table S1**).

Lipoplexes formed with DMCP/**2**/F9-PEG-Lipid appear to be more heavily affected by the presence of siRNA, due to the large increase of average hydrodynamic diameter and PDI revealed by cumulant analysis. To ascertain this effect, a deeper look at the number-weighted size distribution is of valuable help. Actually, this analysis reveals that the enlargement of the average size is due to the presence of two populations of lipoplexes with different numerosity. The most abundant lipoplexes have a size of 60 nm while the larger one, which can be attributed to sporadic aggregation of the smaller lipoplexes, is present at less than 1 %. Even if not fully significant, the slight decrease of lipoplex size with respect to the one of DMCP/**2**/F9-PEG-Lipid can be attributed to the modification of the interfacial properties of the liposome, *i.e.* hydration and distribution of PEG chain, due to the presence of siRNA.

*S4, Detection of the C1858T variant in the PTPN22 gene*

Genomic leukocyte DNA was extracted from whole blood samples of patients by QIAmp DNA blood mini kit (Qiagen, Hilden Germany) according to manufacturer’s guidelines. Polymerase chain reaction (PCR) was carried out with specific primers for exon 14 of the gene *PTPN22* (Gene Bank ID: 26191): forward 5’- gataatgttgcttcaacggaattt -3’ and reverse 5’- cctcaaactcaaggctcacac -3’. The amplification lasted 37 cycles with 58.5 ˚C annealing temperature. PCR sequencing was carried out with the BigDye Terminator v.3.1 Cycle sequencing protocol (Life Technologies, Applied Biosystems, Paisley, Scotland, UK). Products were then purified and sequenced with the Genetic Analyzer 3500 (Applied Biosystems HITACHI system).

*S5, Cell preparation*

PBMC were isolated from sodium heparinized venous blood samples (5-10 ml) using Ficoll-Hypaque (Histopaque, Sigma-Aldrich Chemical Co.) belonging to recruited individuals and then frozen down in liquid nitrogen according to standard protocols^24^.

*S6, Confocal microscopy analysis*

PBMC from healthy donors (HD) were seeded at 1.5 x 10^6^ cells per well in 48-well plates (Falcon) in a final volume of 250 µl of FBS-free RPMI 1640 medium (EuroClone) supplemented with L-glutamine (2 mM) (EuroClone) and treated with L-siRNA or L-siRNA-Sig10L complexes marked with PKH26 (0.5-1 μM with 80, 100 pmols of siRNA), a red‐fluorescent lipophilic membrane dye. After 4.5 h or O/N transfection, cells were harvested, washed in PBS (Euroclone), and fixed with 4 % paraformaldehyde (Sigma-Aldrich Chemical Co.). Fixed cell suspensions were distributed drop wise onto positive charged microscope slides (Super Frost plus, Menzel-Glaser, Germany) and dried at 37 °C. After rehydration in PBS, cells were permeabilized with 0.1 % PBS-Triton X-100 (Sigma-Aldrich Co.) for 5 minutes. After 30 minutes blocking with 5 % bovine serum albumin (BSA, Sigma-Aldrich Co.) in PBS cells were stained with primary mouse anti-human CD3 (Clone UCHT1 BD Biosciences, San Jose, CA, 1:30) incubated for 1 h at RT followed by secondary antibody (Ab) goat anti-mouse Cy-5 conjugate (Invitrogen, 1:100) incubated for 1 h at RT. To counterstain plasma membrane and nuclei, WGA (wheat germ agglutinin) conjugated to Oregon Green 488 (Invitrogen, 1:200) and Hoechst 33342 (Invitrogen, 1 μg/ml) were used respectively. Confocal imaging was performed on an Olympus Fluoview FV1000 confocal microscope equipped with FV10-ASW version 4.1a software, Multi Ar (458±488 and 515 nm), 2× He/Ne (543 and 633 nm), and 405-nm diode lasers, using a 60× (1.42 NA oil) objective. Optical single sections were acquired with a scanning mode format of 1024×1024 pixels, sampling speed of 12.5 ms/pixel (pixel size of 0.1 µm), and Z-reconstructions of serial single optical sections were carried out with an electronic zoom at 2. Fluorochromes unmixing was performed by acquisition of automated-sequential collection of multi-channel images, in order to reduce spectral crosstalk between channels.

*S7, Toxicity assay*

Toxicity evaluation of lipoplexes was assessed by monitoring cell morphology, viability, quantity and quality of cell pellets and quantification of protein extract concentration at the end of the experimental procedure. HD PBMC were seeded at density of 1.5 x 10^6^ cells per well in 48-well plates (Falcon) in a final volume of 250 µl of FBS-free RPMI 1640 medium (EuroClone) supplemented with L-glutamine (2 mM) (EuroClone) and treated with different doses of L-siRNA-Sig10L (with 1.5 % Sig10L, liposome/siRNA ratio 1:1, 80 and 100 pmols of siRNA^17^) complexes labeled with PKH26 (0.1 μM)^4^. After 4.5 h or O/N transfection, cells were harvested with complete medium, centrifuged at 1200 rpm for 5 minutes, washed once in PBS and resuspended in PBS (EuroClone) 2 % FBS. To identify T cell subsets without further selection for CD3+ and CD3- cells, cells were stained for 20 minutes at 4 °C with Brilliant Ultraviolet 737 (BUV737) conjugated mouse anti-human CD3 (Clone UCHT1; 1:40 dilution; BD Biosciences, CA, USA). After incubation, cells were washed once in PBS (EuroClone) 2 % FBS and centrifuged at 1200 rpm for 5 minutes at RT. Subsequently, to detect and quantify dead cells, the blue fluorescent cell impermeable 4′,6-diamidine-2′-phenylindole dihydrochloride dye (DAPI, Invitrogen) was added at a final concentration of 0.2 µM, 5 minutes prior to acquisition of cells by flow-cytometer BD LSR Fortessa X-20 (BD, Sunnyvale, CA). Gating strategy used in FACS analysis is reported in **Figure S2.**


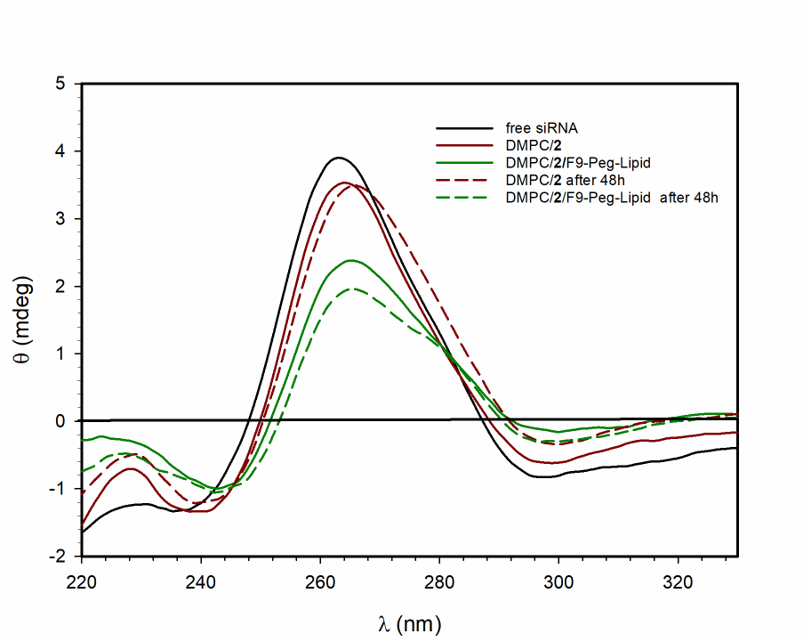


**Figure S1.** CD spectra of: siRNA in Hepes/EDTA (solid black line); DMPC/2/siRNA lipoplexes at 3h from preparation (solid red line) and after 48 h (dashed red line); DMPC/2/F9-PEG-Lipid/siRNA at 3h from preparation (solid green line) and after 48 h (dashed green line). [siRNA]=1.3 µM in all samples. CD spectra were recorded at 20 °C in a 0.5 cm path length cell.


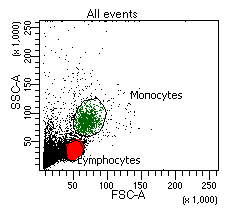


**A**

**B**

**C**


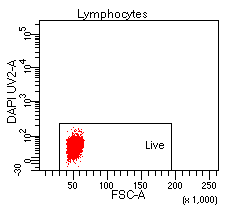

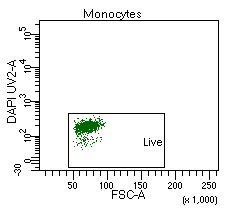


**Figure S2.** Gating strategy used in the flow-cytometry analysis to define lymphocytes and monocytes cell subsets. All cells **(A)**, lymphocytes **(B)** and monocytes **(C)** were gated through morphological parameters and their forward/scatter properties (FSC-A/SSC-A plot). Dead cells were excluded by using the blue fluorescent cell impermeable DAPI dye staining.

**A**

**B**

**Figure S3.** Assessment of LiposiRNA efficacy on target mRNA. Target mRNA levels related to the 4 wild-type *PTPN22* patients upon 48 h **(A)** and 72 h **(B)** of treatment.

**S1 Table**. Data represent the mean ± SD. The asterisk (*) is referred to the less abundant population.

| **Sample** | **2 RH [nm]**  **cumulant method** | **PDI index** | **2 R_H_ [nm]**  **NNLS –**  **Number Weighted analysis** |
| --- | --- | --- | --- |
| DMPC/**2** | 95.1±1.7 | 0.090±0.016 | 66.9±2.5 |
| DMPC/**2**/F9-PEG-Lipid | 122.3±5.4 | 0.185±0.015 | 65.6±9.7 |
| DMPC/**2**/siRNA | 99.1±4.5 | 0.102±0.004 | 66.7±2.9 |
| DMPC/**2**/F9-PEG-Lipid/siRNA | 163±12 | 0.284±0.007 | 60.03±6.2; 250±21* |

**S2 Table.** Demographic, genetic and clinical characteristics of patients selected for the functional assay.

| **Patient** | **Gender** | **Age of disease onset (Years)** | **Age at referral (Years)** | **Duration of Disease (Years)** | **Autoimmune disorders** | ***PTPN22* genotype** | **HbA1c at referral** | **Treatment** |
| --- | --- | --- | --- | --- | --- | --- | --- | --- |
| 1 | M | 4.88 | 11.80 | 6.92 | T1D | 1858C/1858T | **56** | CSII |
| 2 | M | 8.66 | 25.19 | 16.53 | T1D | 1858C/1858T | **54** | CSII |
| 3 | M | 12.65 | 19.11 | 6.45 | T1D | 1858C/1858T | **94** | MDII |
| 4 | M | 7.09 | 16.99 | 9.9 | T1D | 1858C/1858T | **79** | CSII |
| 5 | M | 17.88 | 24.35 | 6.46 | T1D | 1858C/1858T | **60** | CSII |

Glycated hemoglobin (HbA1c, reference values 20.0 - 38.0 mmol/mol). Pathological values are highlighted in bold. Multiple Daily Insulin Injection (MDII), Continuous Subcutaneous Insulin Infusion (CSII).

**References**

1. Koppel DE. Analysis of Macromolecular Polydispersity in Intensity Correlation Spectroscopy: The Method of Cumulants. J Chem Phys (1972) 57:4814–20. doi: 10.1063/1.1678153.
2. Lawson CL, Hanson RJ. Solving Least Squares Problems Prentice-Hall Vol. xii + 340. New Jersey: Englewood Cliffs (1974).
3. Aleandri S, Bonicelli MG, Bordi F, Casciardi S, Diociaiuti M, Giansanti L, et al. How Stereochemistry Affects the Physicochemical Features of Gemini Surfactant Based Cationic Liposomes. Soft Matter (2012) 8:5904–15. doi: 10.1039/c2sm25193k.
4. Song X, Ren Y, Zhang J, Wang G, Han X, Zheng W, et al. Targeted Delivery of Doxorubicin to Breast Cancer Cells by Aptamer Functionalized DOTAP/ DOPE Liposomes. Oncol Rep (2015) 34:1953–60. doi: 10.3892/or.2015.4136.
